# Supplementary material for: The receptor kinase SRF3 coordinates iron-level and flagellin dependent defense and growth responses in plants
Source: Nat Commun. 2022 Aug 1;13:4445. doi: 10.1038/s41467-022-32167-6 (PMC9343624; doi:10.1038/s41467-022-32167-6)
Supplement: Supplementary file 1 — Supplementary Information [file 41467_2022_32167_MOESM1_ESM.pdf]

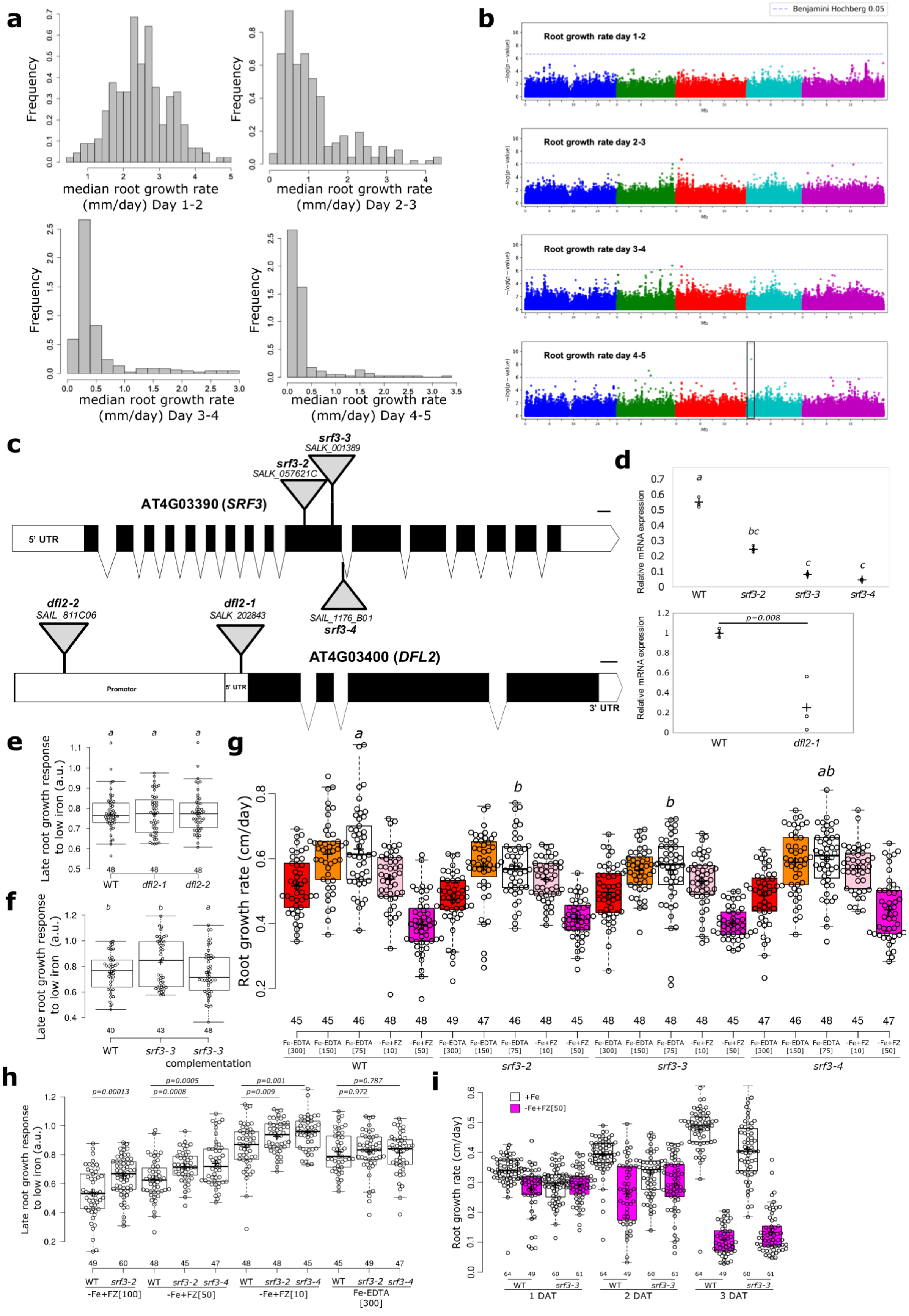

a
Frequency
median root growth rate (mm/day) Day 1-2
median root growth rate (mm/day) Day 2-3
median root growth rate (mm/day) Day 3-4
median root growth rate (mm/day) Day 4-5
b
Benjamini Hochberg 0.05
Root growth rate day 1-2
Root growth rate day 2-3
Root growth rate day 3-4
Root growth rate day 4-5
−log(p − value)
Mb
c
srf3-3
SALK_001389
srf3-2
SALK_057621C
AT4G03390 (SRF3)
5' UTR
SAIL_1176_B01
srf3-4
dfl2-2
SAIL_811C06
dfl2-1
SALK_202843
AT4G03400 (DFL2)
Promotor
5' UTR
3' UTR
d
Relative mRNA expression
a
bc
c
c
WT
srf3-2
srf3-3
srf3-4
p=0.008
WT
dfl2-1
e
Late root growth response to low iron (a.u.)
a
a
a
48
48
48
WT
dfl2-1
dfl2-2
f
Late root growth response to low iron (a.u.)
b
b
a
40
43
48
WT
srf3-3
srf3-3 complementation
g
Root growth rate (cm/day)
a
b
b
ab
45 45 46 48 48 49 47 46 48 45 48 48 48 48 45 47 46 48 45 47
Fe-EDTA [300]
Fe-EDTA [150]
Fe-EDTA [75]
-Fe+FZ [10]
-Fe+FZ [50]
WT
srf3-2
srf3-3
srf3-4
h
Late root growth response to low iron (a.u.)
p=0.00013
p=0.0005
p=0.0008
p=0.001
p=0.009
p=0.787
p=0.972
49 60 48 45 47 48 48 45 45 49 47
WT srf3-2 WT srf3-2 srf3-4 WT srf3-2 srf3-4 WT srf3-2 srf3-4
-Fe+FZ[100]
-Fe+FZ[50]
-Fe+FZ[10]
Fe-EDTA [300]
i
+Fe
-Fe+FZ[50]
Root growth rate (cm/day)
64 49 60 61 64 49 60 61 64 49 60 61
WT
srf3-3
1 DAT
2 DAT
3 DAT

Supplementary Figure 1. Natural variation of Arabidopsis accessions grown under–Fe and characterization of GWAS candidate genes.

(a) Histograms of median root growth rates (mm) of accessions grown on Fe deficient growth conditions (root growth rate day 1-2, root growth rate day 2-3, root growth rate day 3-4, root growth rate day 4-5). x-axis: median root growth rate in mm; y-axis: frequency. **(b)** Manhattan plots depicting genome wide SNP associations for median root growth rate on Fe deficient growth conditions. The chromosomes are depicted in different colors. The horizontal blue dash-dot line corresponds to a nominal 0.05 significance threshold after Benjamini-Hochberg Correction. Black box indicates the significantly associated region in close proximity to *SRF3* gene. x-axis: chromosomal position of SNP; y-axis: -$\log_{10}$(*p*-value). **(c)** Cartoon showing the genomic structures of (a) AT4G03390 and (b) AT4G03400 and the T-DNA insertion sites***.*** Black boxes indicate exons. Scale bar: 100 bp. Gene models were generated by Exon-Intron graphic maker (http://wormweb.org/exonintron). **(d)** Transcript analysis of AT4G03390 in *srf3-2, srf3-3* and *srf3-4* and transcript analysis of AT4G03400 in *at4g03400.* **(e, f)** Root growth response doing transfer assay under low iron levels supplemented with 100 µM ferrozine with WT, SALK_202843 and SAIL811_C06 **(E)** and WT, *srf3-3*, *srf3-3* complementation line **(f)**. One-way ANOVA follows by a post-hoc Tukey HSD test, letters indicate statistical differences ($p<0.05$). **(g)** Graph of the root growth rate expressed in cm/day exposed to low iron levels for 3 days after transfer to media provided by 100 µM, 50 µM or 10 µM of ferrozine and 300 µM of Na-Fe-EDTA on wild type (WT) and *srf3-2*, *srf3-3* and *srf3-4*. Independent two ways student test ($p<0.05$), n.s. non-significant. **(h)** Graph of the root growth response to low iron levels for 3 days provided by 100 µM, 50 µM or 10 µM of ferrozine and 300 µM of Na-Fe-EDTA on wild type (WT) and *srf3-2* and *srf3-4*. Note that we used the root growth response all along the study to provide more clarity to the results and keep the figure more readable and interpretable. Two ways student test ($p<0.05$, n.s. = non-significant). **(i)** Graph of the root growth rate expressed in cm/day exposed to sufficient iron level or low iron levels containing 50 µM of ferrozine for 3 days after transfer in WT and *srf3-3* genotypes. For boxplots, circles indicate a single biological replicate and the number below each box specifies the number of replicates, horizontal black bars indicate the median, the black cross represents the mean, box represents the interquartile range and the hinges the min and max whiskers.

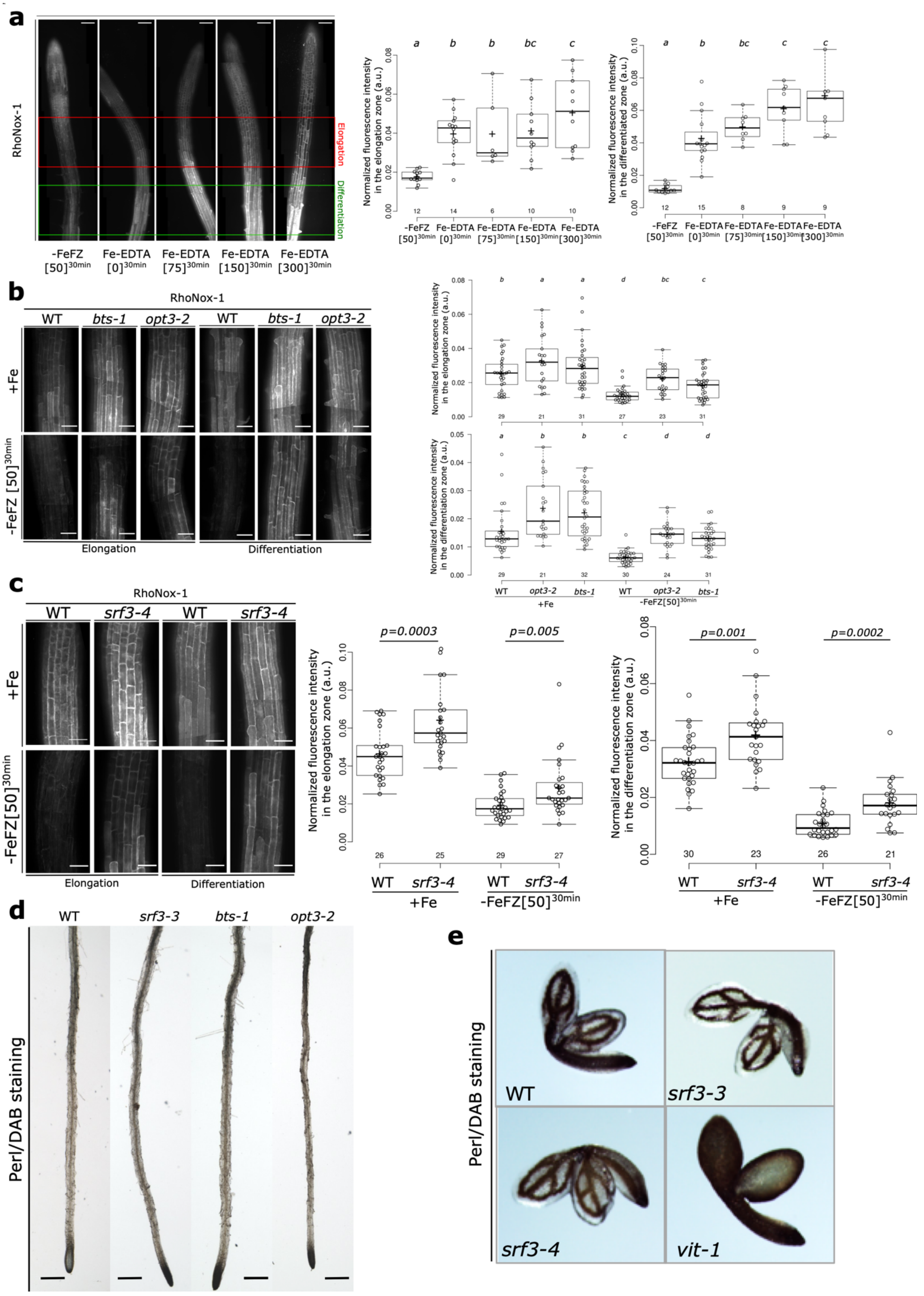
a
RhoNox-1
Elongation
Differentiation
-FeFZ [50]30min
Fe-EDTA [0]30min
Fe-EDTA [75]30min
Fe-EDTA [150]30min
Fe-EDTA [300]30min
Normalized fluorescence intensity in the elongation zone (a.u.)
Normalized fluorescence intensity in the differentiated zone (a.u.)
a b b bc c
a b bc c c
12 14 6 10 10
12 15 8 9 9
b
RhoNox-1
WT bts-1 opt3-2 WT bts-1 opt3-2
+Fe
-FeFZ [50]30min
Elongation
Differentiation
Normalized fluorescence intensity in the elongation zone (a.u.)
b a a d bc c
29 21 31 27 23 31
Normalized fluorescence intensity in the differentiation zone (a.u.)
a b b c d d
29 21 32 30 24 31
WT opt3-2 bts-1 WT opt3-2 bts-1
+Fe
-FeFZ[50]30min
c
RhoNox-1
WT srf3-4 WT srf3-4
+Fe
-FeFZ[50]30min
Elongation
Differentiation
Normalized fluorescence intensity in the elongation zone (a.u.)
p=0.0003
p=0.005
26 25 29 27
WT srf3-4 WT srf3-4
+Fe
-FeFZ[50]30min
Normalized fluorescence intensity in the differentiation zone (a.u.)
p=0.001
p=0.0002
30 23 26 21
WT srf3-4 WT srf3-4
+Fe
-FeFZ[50]30min
d
WT srf3-3 bts-1 opt3-2
Perl/DAB staining
e
Perl/DAB staining
WT
srf3-3
srf3-4
vit-1

Supplementary Figure 2. Characterization of RhoNox-1 staining and evaluation of iron content in embryos of WT, *srf3* mutants and *vit-1*. (a) Left panel: confocal images epidermal root cells of 5 day old seedling of WT stained with RhoNox-1 pretreated with 50µM, 0 µM of FerroZine (FZ) and 75, 150 and 300 µM of Na-Fe-EDTA for 30 minutes. Red box indicates the region where the fluorescence intensity has been quantified in the differentiation zone while green box indicates the elongation zone. Scale bars, 100µm. Middle panel: the related fluorescence intensity quantification in the elongation zone. Right panel: the related fluorescence intensity quantification in the differentiation zone. One-way ANOVA, follows by a post-hoc Tukey HSD test, letters indicate statistical differences ($p<0.05$). **(b)** Left panel: confocal images of elongation and differentiated epidermal root cells stained with RhoNox-1, WT, *bts-1* and *opt3-2* in sufficient (+Fe), upper panel, and in low iron levels (-Fe) with 50µM of ferrozine for 30 minutes. Scale bars, 50µm. Upper right panel: the related quantification of the normalized fluorescence intensity in the elongation zone. Lower right panel: the related quantification of the normalized fluorescence intensity in the differentiation zone. One-way ANOVA, follows by a post-hoc Tukey HSD test, letters indicate statistical differences ($p<0.05$). **(c)** Left panel: confocal images of elongation and differentiated epidermal root cells stained with RhoNox-1, WT and *srf3-4* in sufficient (+Fe) and in low iron levels (-Fe) supplemented with 50 µM of ferrozine for 30 minutes. Scale bars, 50µm. Middle panel: related quantification of the normalized fluorescence intensity in the elongation zone. Right panel: related quantification of the normalized fluorescence intensity in the differentiated zone. One-way ANOVA followed by a post-hoc Tukey HSD test, letters indicate statistical differences ($p<0.05$). **(d)** Root of WT, *srf3-3*, *bts-1* and *opt3-2* (positive control) stained with Perls/DAB method **(e).** WT, *srf3-3*, *srf3-4* and *vit-1* (positive control) dry seed embryos were dissected and stained with Perls/DAB method. For boxplots, circles indicate a single biological replicate and the number below each box specifies the number of replicates, horizontal black bars indicate the median, the black cross represents the mean, box represents the interquartile range and the hinges the min and max whiskers.

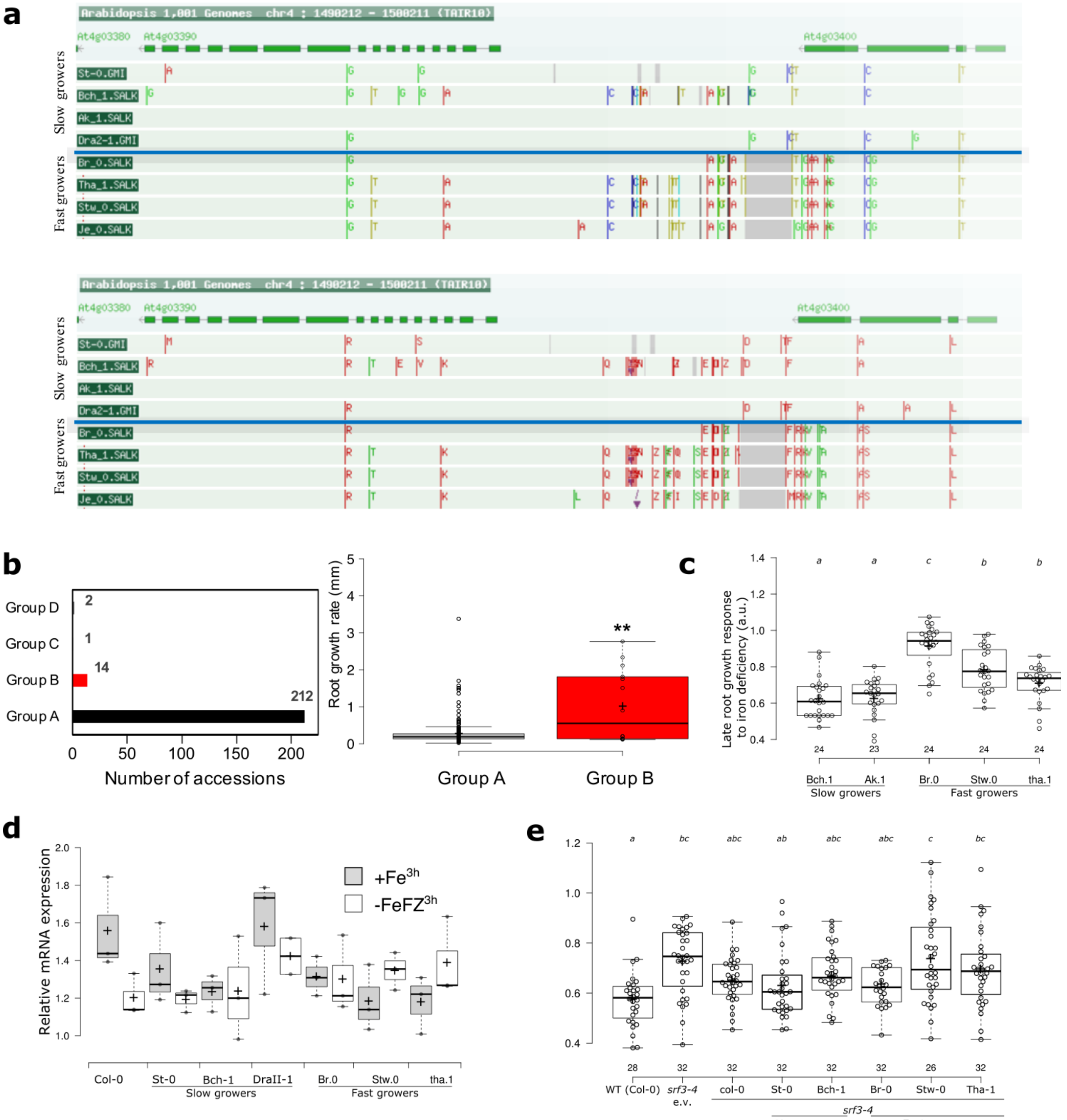

a
Arabidopsis 1,001 Genomes chr4 : 1490212 - 1500211 (TAIR10)
At4g03380
At4g03390
At4g03400
Slow growers
Fast growers
St-0.GMI
Bch_1.SALK
Ak_1.SALK
Dra2-1.GMI
Br_0.SALK
Tha_1.SALK
Stw_0.SALK
Je_0.SALK
b
Group D
Group C
Group B
Group A
2
1
14
212
0
50
100
150
200
Number of accessions
Root growth rate (mm)
**
Group A
Group B
c
Late root growth response to iron deficiency (a.u.)
a
a
c
b
b
24
23
24
24
24
Bch.1
Ak.1
Br.0
Stw.0
tha.1
Slow growers
Fast growers
d
Relative mRNA expression
+Fe3h
-FeFZ3h
Col-0
St-0
Bch-1
DraII-1
Br.0
Stw.0
tha.1
Slow growers
Fast growers
e
a
bc
abc
ab
abc
abc
c
bc
28
32
32
32
32
32
26
32
WT (Col-0)
srf3-4 e.v.
col-0
St-0
Bch-1
Br-0
Stw-0
Tha-1
srf3-4
Slow growers
Fast growers

Supplementary Figure 3. Sequence variation around the SRF3 locus and SRF3 allele associated gene expression and root growth. **(a)** Sequence polymorphisms (top: SNP; bottom: amino acid) surrounding the SRF locus in four representative accessions displaying slow root growth and fast root growth on -Fe respectively (data from: http://signal.salk.edu/atg1001/3.0/gebrowser.php). **(b)** Left panel: distribution of marker SNPs highlighted in red color in haplogroup A (major), B (minor), C (minor) and D (minor). Bar plot shows frequency of haplogroups A, B, C and D in 231 accessions. Right panel: box plots for root growth rate in Group A (major) and Group B (minor) accessions. Asterisks indicate significant difference with Tukey's HSD comparison (p-value < 0.05). **(c)** Late root growth response to low iron levels after transfer with extreme accession found in the GWAS. One-way ANOVA follows by a post-hoc Tukey HSD test, letters indicate statistical differences (p<0.05). **(d)** Relative expression level of SRF3 in Col-0, St-0, Bch-1, DraII-1, Br-0, Stw-0 and Tha-1 at T0, in mock treatment after 3 hours in low iron with 100 µM of ferrozine. **(e)** Box plot of the root growth response to low iron levels for 3 days of Col-0, *srf3-4* and allelic complementation lines of St-0, Bch-1, Br-0, Stw-0 and Tha-1. One-way ANOVA follows by a post-hoc Tukey HSD test, letters indicate statistical differences (p<0.05). For boxplots, circles indicate a single biological replicate and the number below each box specifies the number of replicates, horizontal black bars indicate the median, the black cross represents the mean, box represents the interquartile range and the hinges the min and max whiskers.

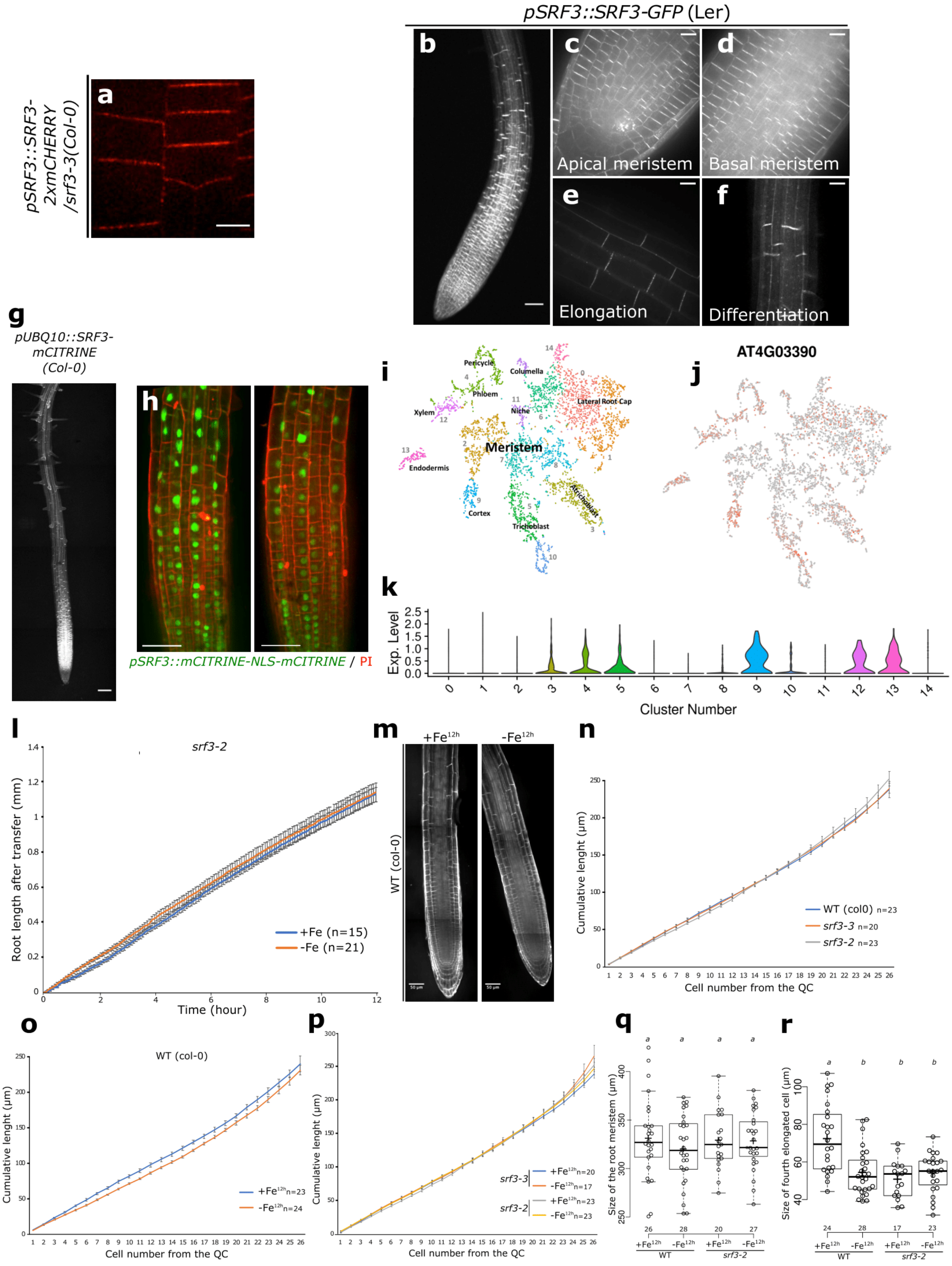
pSRF3::SRF3-GFP (Ler)
a
pSRF3::SRF3-2xmCHERRY/srf3-3(Col-0)
b
c
d
Apical meristem
Basal meristem
e
f
Elongation
Differentiation
g
pUBQ10::SRF3-mCITRINE (Col-0)
h
pSRF3::mCITRINE-NLS-mCITRINE / PI
i
Pericycle
Columella
Phloem
Xylem
Niche
Lateral Root Cap
Meristem
Endodermis
Cortex
Trichoblast
Atrichoblast
j
AT4G03390
k
Exp. Level
Cluster Number
l
srf3-2
Root length after transfer (mm)
Time (hour)
+Fe (n=15)
-Fe (n=21)
m
+Fe12h
-Fe12h
WT (col-0)
n
Cumulative lenght (µm)
Cell number from the QC
WT (col0) n=23
srf3-3 n=20
srf3-2 n=23
o
WT (col-0)
Cumulative lenght (µm)
Cell number from the QC
+Fe12h n=23
-Fe12h n=24
p
Cumulative lenght (µm)
Cell number from the QC
srf3-3
+Fe12h n=20
-Fe12h n=17
srf3-2
+Fe12h n=23
-Fe12h n=23
q
Size of the root meristem (µm)
26
28
20
27
+Fe12h
-Fe12h
WT
srf3-2
r
Size of fourth elongated cell (µm)
24
28
17
23
+Fe12h
-Fe12h
WT
srf3-2

Supplementary Figure 4. SRF3 transcription and translation in the transition-elongation zone. (a) Confocal image of root epidermal cells in the transition-elongation zone of *pSRF3::SRF3-2xmCHERRY-4xmyc* in the *srf3-3* mutant that is in the Col-0 background (*srf3-3* comp). This has been observed on at least 20 roots throughout three independent experiments. Scale bar, 10µm. **(b,c, d, e, f)** Confocal images of 5 day-old seedlings expressing *pSRF3::SRF3-GFP* in Ler-0 background **(b)** (scale bar, 50µm), apical meristem **(c),** basal meristem **(d),** elongation **(e)** and differentiated tissue **(f)**. (Scale bars, 10µm). **(g)** Confocal image of root of 5 day-old seedling expressing *UBQ10::SRF3-mCITRINE* (*SRF3-OX*) in Col-0 background. This has been observed on at least 20 roots throughout three independent experiments. Scale bar 50µm. (**h**) Confocal images of 5 day-old seedlings expressing *pSRF3::mCITRINE-NLS-mCITRINE* counterstained with propidium iodide (PI). Scale bars, 50µm. This has been observed on at least 20 roots throughout three independent experiments. **(i)** Cell-type annotation of clusters for t-SNE plot in (j). **(j)** t-SNE plot with cells highlighted in red that express *SRF3* (AT4G03390). **(k)** Violin-plot depicting the distribution of *SRF3* expression levels in cluster shown in (i,j). Y-axis: gene expression level distribution of *SRF3* within each cluster. X-axis: Proportion of cells showing a given *SRF3* expression value. **(l)** Time lapse analysis of root growth of WT and *srf3-2* under sufficient and low iron levels. Error bars indicate standard error of the mean (SEM). **(m)** Confocal images of 5-day old seedlings stained with propidium iodide in WT under sufficient and low iron levels for 12 hours. This has been observed on at least 20 roots throughout three independent experiments. Scale bars, 50µm. **(n,o,p)** Cumulative root cell length of 6 day old seedlings measured starting from the quiescent center after 12 hours under sufficient iron levels in WT and *srf3* mutants (**n**), under sufficient and low iron levels in WT (col-0) (**o**) and srf3 mutants (**p**). Error bars indicate standard error of the mean (SEM). (**q**) Root meristem length in WT and *srf3-2* after 12 hours under iron sufficient condition or low iron levels. One-way ANOVA followed by a post-hoc Tukey HSD test, letters indicate statistical differences ($p<0.05$). (**r**) Size of the fourth elongated cell. One-way ANOVA followed by a post-hoc Tukey HSD test, letters indicate statistical differences ($p<0.05$). For boxplots, circles indicate a single biological replicate and the number below each box specifies the number of replicates, horizontal black bars indicate the median, the black cross represents the mean, box represents the interquartile range and the hinges the min and max whiskers.

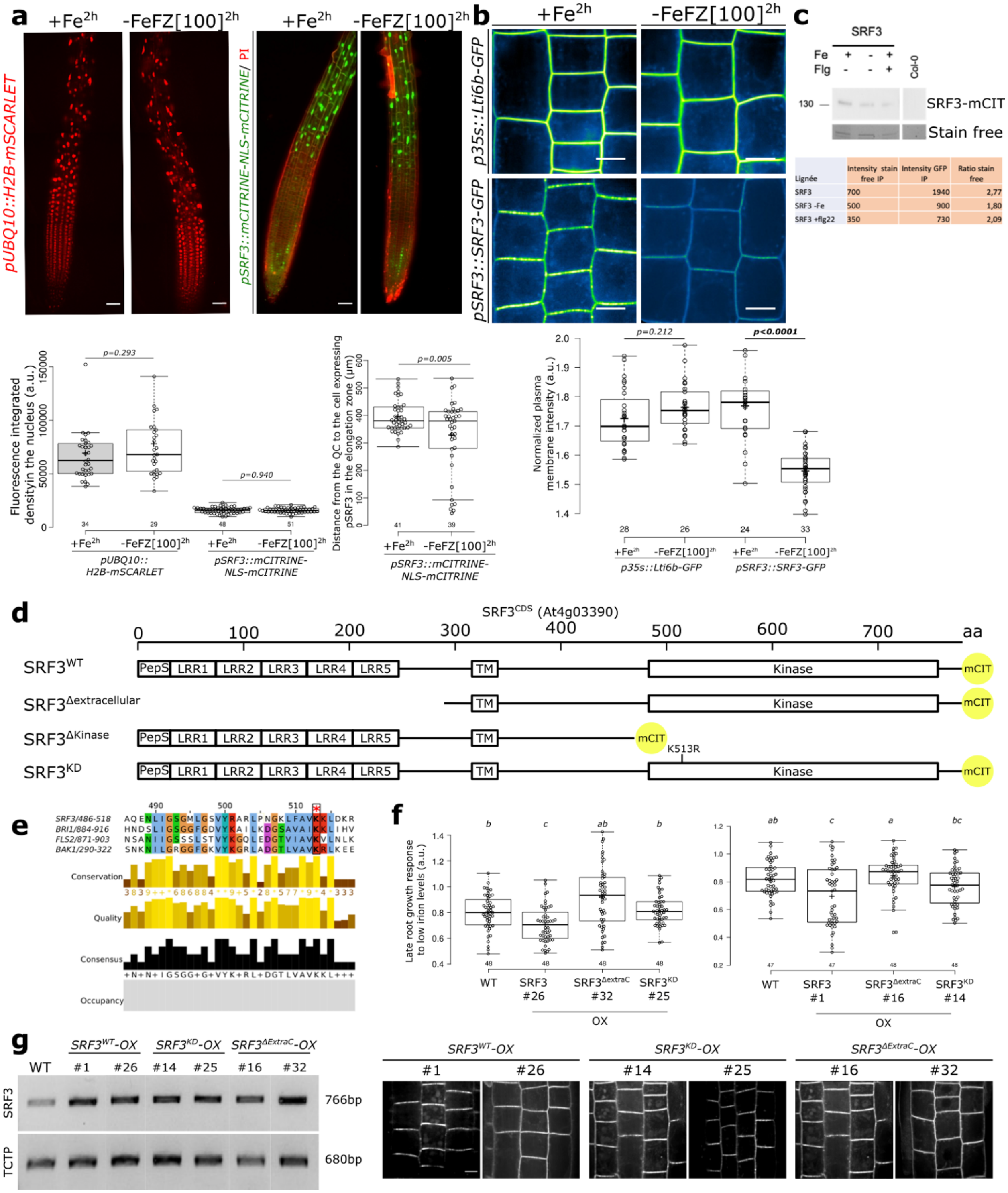

Lignée	Intensity stain free IP	Intensity GFP IP	Ratio stain free
SRF3	700	1940	2,77
SRF3 -Fe	500	900	1,80
SRF3 +flg22	350	730	2,09

Supplementary Figure 5. SRF3 protein level and protein domain dependent root growth regulation. (a) Upper panel: Confocal images of root tips of 5 day old seedlings expressing *pUBQ10::H2B-mSCARLET* (left) and *pSRF3::mCITRINE-NLS-mCITRINE* (right) and cells were stained by propidium iodide (PI) under mock (+Fe) and under low iron levels generated with 100 µM of ferrozine (-FeFZ[100]) for 2 hours. This has been observed on at least 29 roots throughout three independent experiments. Scale bars, 50µm. (Lower panel) quantification of the nuclear fluorescence integrated density (lower left) and of the distance from the QC to the cell expressing pSRF3 in the elongation zone in µm (lower right). Independent two-ways Student's T-test ($p<0.05$), n.s. non-significant. (**b)** Upper panel: Confocal images of root epidermis of 5 days old seedling expressing *p35s::Lti6b-GFP* and *pSRF3::SRF3-GFP* (Ler-0 background) under mock (+Fe) and under low iron levels provided by 100 µM of ferrozine (FZ) for two hours. This has been observed on at least 24 roots throughout three independent experiments. Scale bar, 10µm. Lower panel: quantification of the normalized plasma membrane intensity. Independent two-ways Student's T-test ($p<0.05$), n.s. non-significant. (**c**) Western blot of root samples of WT and UBQ10::SRF3-mCITRINE after 4 hours of treatment with -Fe and 1 µM flg22. Upper blot: α-GFP evaluating mCITRINE levels, lower blot: α-Tubulin. Lower panel: Quantification of western blot signals. **(d)** Representation of SRF3 CDS and the utilized truncated and mutated versions. **(e)** Alignment of SRF3, BRI1, FLS2 and BAK1 ATP binding pockets. **(f)** Late root growth response to low iron levels of WT and overexpressing lines of SRF3 CDS full length as well as truncated version and mutated version in Col-0 background. Left, one-way ANOVA followed by a post-hoc Tukey HSD test, letters indicate statistical differences ($p<0.05$), right, two-ways Kruskal-Wallis coupled with post hoc Steel-Dwass-Critchlow-Fligner procedure was performed ($p<0.05$) (**g**) RT-PCR using SRF3 primers in two different insertion lines of $SRF3^{WT}$ (UBQ10::SRF3-mCITRINE), $SRF3^{KD}$ (UBQ10::$SRF3^{KD}$-mCITRINE), $SRF3^{KD}$ (UBQ10::$SRF3^{KD}$-mCITRINE), $SRF3^{\Delta ExtraC}$ (UBQ10::SRF3 $^{\Delta ExtraC}$-mCITRINE) and the related confocal images of root epidermal cells in the transition elongation zone of 5 day old seedlings. This has been observed on at least 20 roots throughout three independent experiments. For boxplots, circles indicate a single biological replicate and the number below each box specifies the number of replicates, horizontal black bars indicate the median, the black cross represents the mean, box represents the interquartile range and the hinges the min and max whiskers.

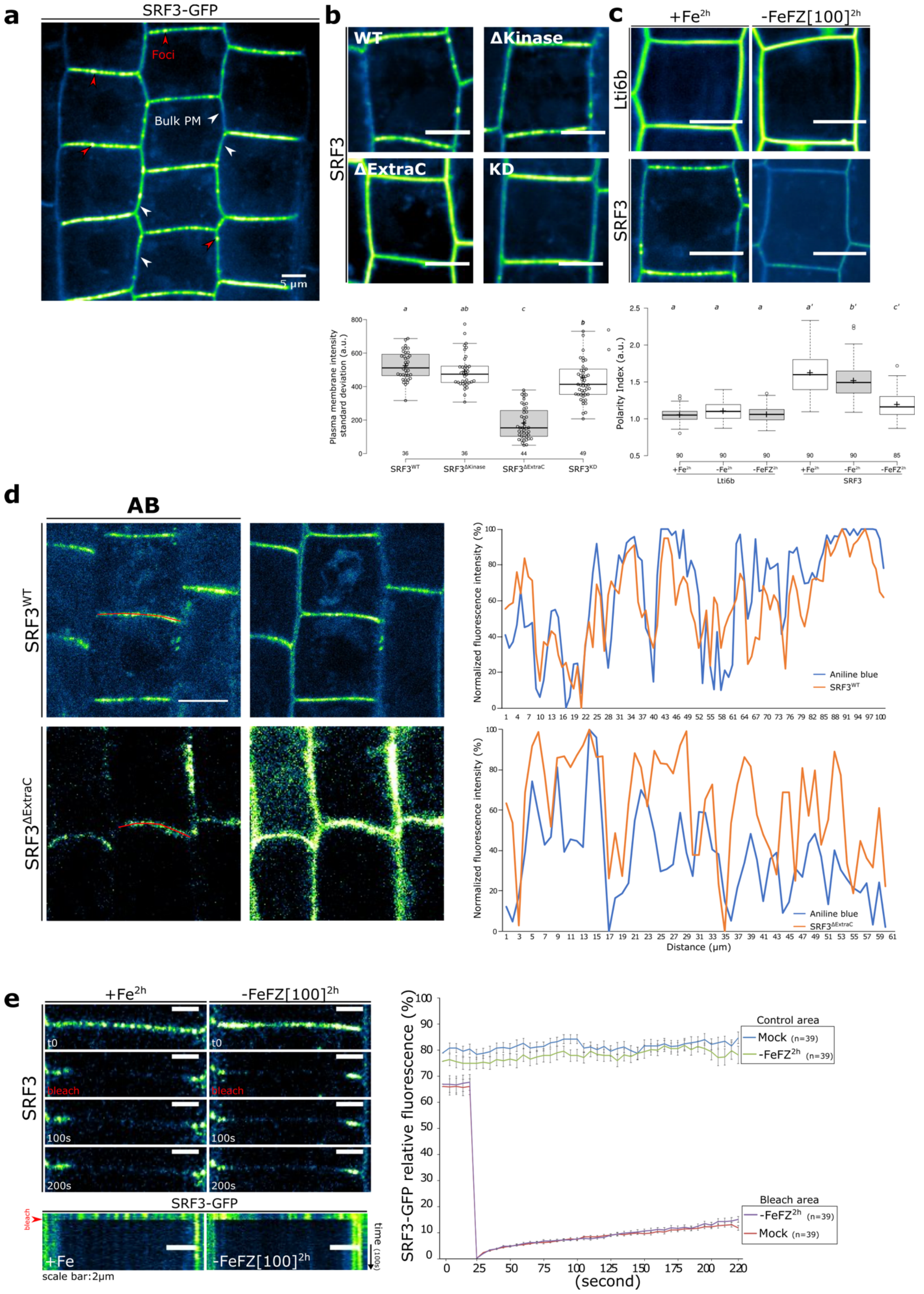

a
SRF3-GFP
Foci
Bulk PM
5 µm
b
WT
ΔKinase
ΔExtraC
KD
SRF3
c
+Fe2h
-FeFZ[100]2h
Lti6b
SRF3
Plasma membrane intensity standard deviation (a.u.)
SRF3WT
SRF3ΔKinase
SRF3ΔExtraC
SRF3KD
Polarity Index (a.u.)
+Fe2h
-Fe2h
-FeFZ2h
Lti6b
SRF3
d
AB
SRF3WT
SRF3ΔExtraC
Normalized fluorescence intensity (%)
Aniline blue
SRF3WT
SRF3ΔExtraC
Distance (µm)
e
+Fe2h
-FeFZ[100]2h
SRF3
t0
bleach
100s
200s
SRF3-GFP
+Fe
-FeFZ[100]2h
time (100s)
scale bar:2µm
SRF3-GFP relative fluorescence (%)
(second)
Control area
Mock (n=39)
-FeFZ2h (n=39)
Bleach area
-FeFZ2h (n=39)
Mock (n=39)

Supplementary Figure 6. SRF3 is removed from the two PM-associated subpopulations under low iron. (a) Confocal images of root epidermal cells in the elongation zone of 5 day old seedlings expressing *pSRF3::SRF3-GFP*. Red arrows indicate examples of foci and white arrows bulk plasma membrane (Bulk PM). This has been observed on at least 30 roots throughout three independent experiments. Scale bar, 5µm. **(b)** Left panel: confocal images of root epidermis of 5 day old seedling expressing *pUBQ10::SRF3^{WT}-mCITRINE*, *pUBQ10::SRF3*$^{\Delta Kinase}$*-mCITRINE ,pUBQ10::SRF3*$^{\Delta ExtraC}$*-mCITRINE, pUBQ10::SRF3^{KD}-mCITRINE* (SRF3^{WT}, SRF3$^{\Delta Kinae}$, SRF3$^{\Delta ExtraC}$ and SRF3^{KD}). Scale bars, 10µm. Right panel: related quantification of the mean standard deviation of the intensity mean at the apical-basal side of the cell. One-way ANOVA followed by a post-hoc Tukey HSD test, letters indicate statistical differences ($p<0.05$). **(c)** Left panel: Confocal images of root epidermal cells in the elongation zone of 5 day old seedling expressing *p35s::Lti6b-GFP* (Lti6b) and *pSRF3::SRF3-GFP* (SRF3) under mock (+Fe) and under low iron media provided or not by 100 µM of ferrozine (-FeFZ[100]) for two hours. Scale bar, 5µm. Right panel related quantification of polarity index. One-way ANOVA followed by a post-hoc Tukey HSD test, letters indicate statistical differences ($p<0.05$). **(d)** Left panel: Confocal images of 5 days old seedlings in the transition-elongation zone of plants expressing SRF3^{WT} (UBQ10::SRF3-mCITRINE) and SRF3$^{\Delta ExtraC}$ (UBQ10::SRF3 $^{\Delta ExtraC}$-mCITRINE) roots stained with the callose maker Aniline Blue. This has been observed on at least 5 roots throughout two independent experiments; right panel: quantified signal intensity of both channels from the apical basal part of the cell. Red line on the left image indicates where the scan line has been traced. Scale bars, 10µm. **(e)** Upper left panel: confocal images of *pSRF3::SRF3-GFP* (SRF3 in Ler-0 background) of 5 day old seedlings during FRAP experiment in WT in mock and low iron media supplemented with 100 µM of ferrozine. Lower left panel: The resulting kymograph (time scale 15 seconds). Scale bar, 2µm. Right panel: *pSRF3::SRF3-GFP* fluorescence intensity at the plasma membrane during FRAP analyses in the different conditions. Error bars indicate standard error of the mean (SEM).

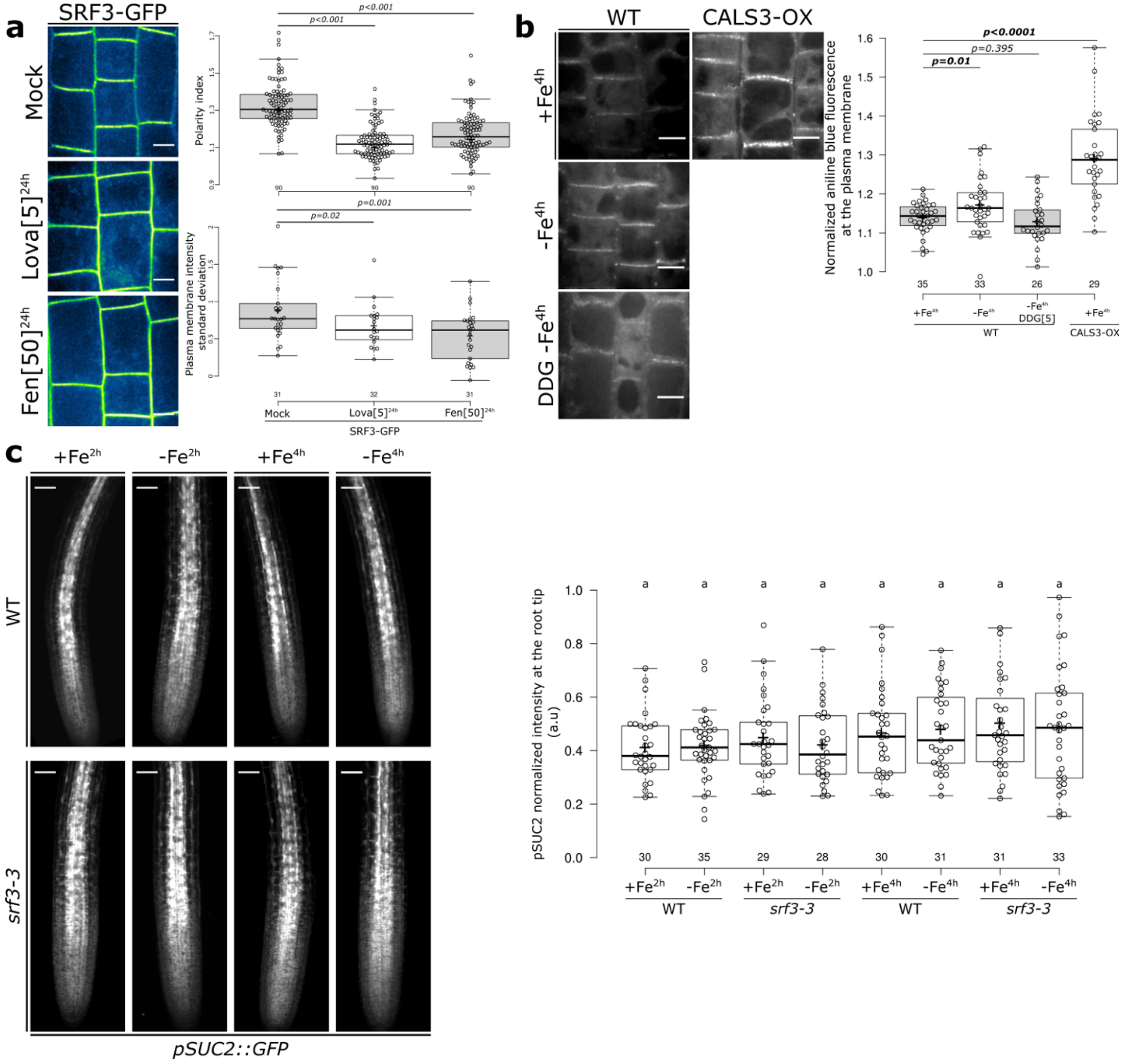
a
SRF3-GFP
Mock
Lova[5]24h
Fen[50]24h
p<0.001
p<0.001
Polarity index
90
90
90
p=0.001
p=0.02
Plasma membrane intensity standard deviation
31
32
31
Mock
Lova[5]24h
Fen[50]24h
SRF3-GFP
b
WT
CALS3-OX
+Fe4h
-Fe4h
DDG -Fe4h
p<0.0001
p=0.395
p=0.01
Normalized aniline blue fluorescence at the plasma membrane
35
33
26
29
+Fe4h
-Fe4h
-Fe4h DDG[5]
+Fe4h
WT
CALS3-OX
c
+Fe2h
-Fe2h
+Fe4h
-Fe4h
WT
srf3-3
pSUC2::GFP
a
pSUC2 normalized intensity at the root tip (a.u)
30
35
29
28
30
31
31
33
+Fe2h
-Fe2h
+Fe2h
-Fe2h
+Fe4h
-Fe4h
+Fe4h
-Fe4h
WT
srf3-3
WT
srf3-3

Supplementary Figure 7. SRF3 is a negative regulator of callose synthase but does not impact cell-to-cell communication. (a) Left panel: confocal images of root epidermal cells of 5 day old seedling expressing *pSRF3::SRF3-GFP* upon 24 hour treatment with mock solution, Lovastin (Lova) and Fenpropimorph (Fen). Scale bar, 10µm. Right panel: related quantification of the polarity index and the standard deviation of the mean intensity at the apical basal part of the cell. Independent two-ways Student's T-test ($p<0.05$), n.s. non-significant. **(b)** Left panel: confocal images of root epidermal cells in the elongation zone of 5 day old seedling stained with aniline blue (AB) in the indicated genotypes, WT and 35S::GFP-CALS3 under mock (+Fe) and under low iron media for four hours with or without 2-deoxy-d-glucose (DDG) Scale bar, 10µm. Right panel: the related quantification of normalized plasma membrane intensity. Independent two-ways Student's T-test ($p<0.05$), n.s. non-significant. **(c)** Confocal images of root tips of 5 day old seedling expressing *pSUC2::GFP* in WT and *srf3-3* under sufficient (+Fe) and low (-Fe) iron conditions and the related quantification [ANOVA with post-hoc Tukey test; Letters: statistical differences ($p<0.05$)]. Scale bars, 50µm. For boxplots, circles indicate a single biological replicate and the number below each box specifies the number of replicates, horizontal black bars indicate the median, the black cross represents the mean, box represents the interquartile range and the hinges the min and max whiskers.

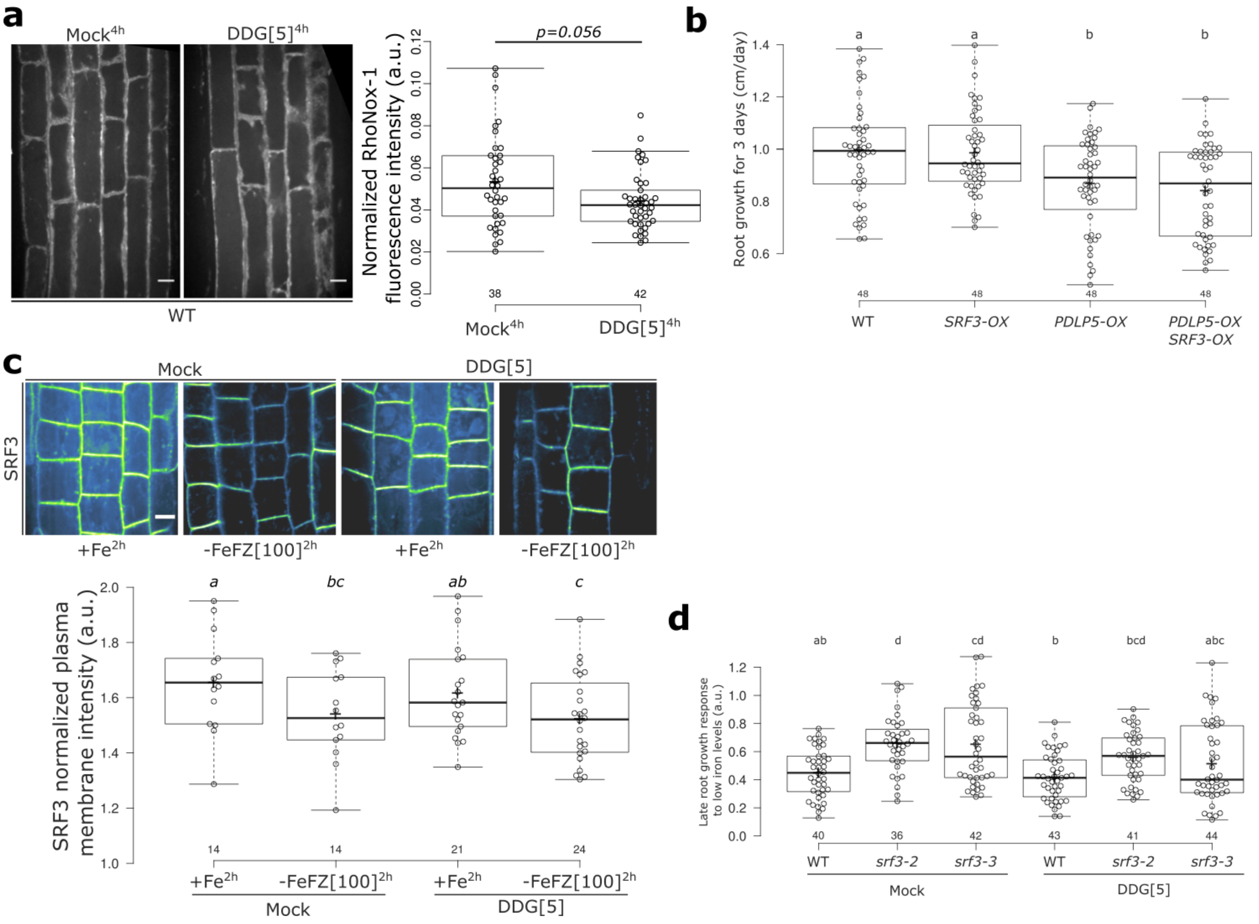

Supplementary Figure 8. SRF3 acts upstream of callose synthase to regulate root growth. (a) Left: confocal images of roots stained with RhoNOX-1 treated without or with DDG. Right: quantification of RhoNOX-1 signal intensity. Independent two-ways Student's T-test ($p<0.05$), n.s. non-significant. **(b)** Quantification of the mean root growth rate for 3 days WT, *UBQ10::SRF3-mCITRINE* (*SRF3-OX), 35S::PDLP5-GFP (PDLP5-OX)* and *PDLP5-GFPxSRF3-OX.* One-way ANOVA followed by a post-hoc Tukey HSD test, letters indicate statistical differences ($p<0.05$). **(c)** Upper panel: confocal images of root epidermal cells in the elongation zone of 5 day old seedlings expressing *pUBQ10::SRF3-mCITRINE* in iron sufficient and deficient media supplemented with 100 µM of ferrozine for 2 hours in presence or absence of DDG. Scale bar, 10µm. Lower panel: related quantification of the normalized plasma membrane intensity. One-way ANOVA followed by a post-hoc Tukey HSD test, letters indicate statistical differences ($p<0.05$). **(d)** Graph representing the root growth response to low iron levels for 3 days with or without 2-deoxy-d-glucose (DDG) in WT, *srf3-2* and *srf3-3.* One-way ANOVA followed by a post-hoc Tukey HSD test, letters indicate statistical differences ($p<0.05$). For boxplots, circles indicate a single biological replicate and the number below each box specifies the number of replicates, horizontal black bars indicate the median, the black cross represents the mean, box represents the interquartile range and the hinges the min and max whiskers.

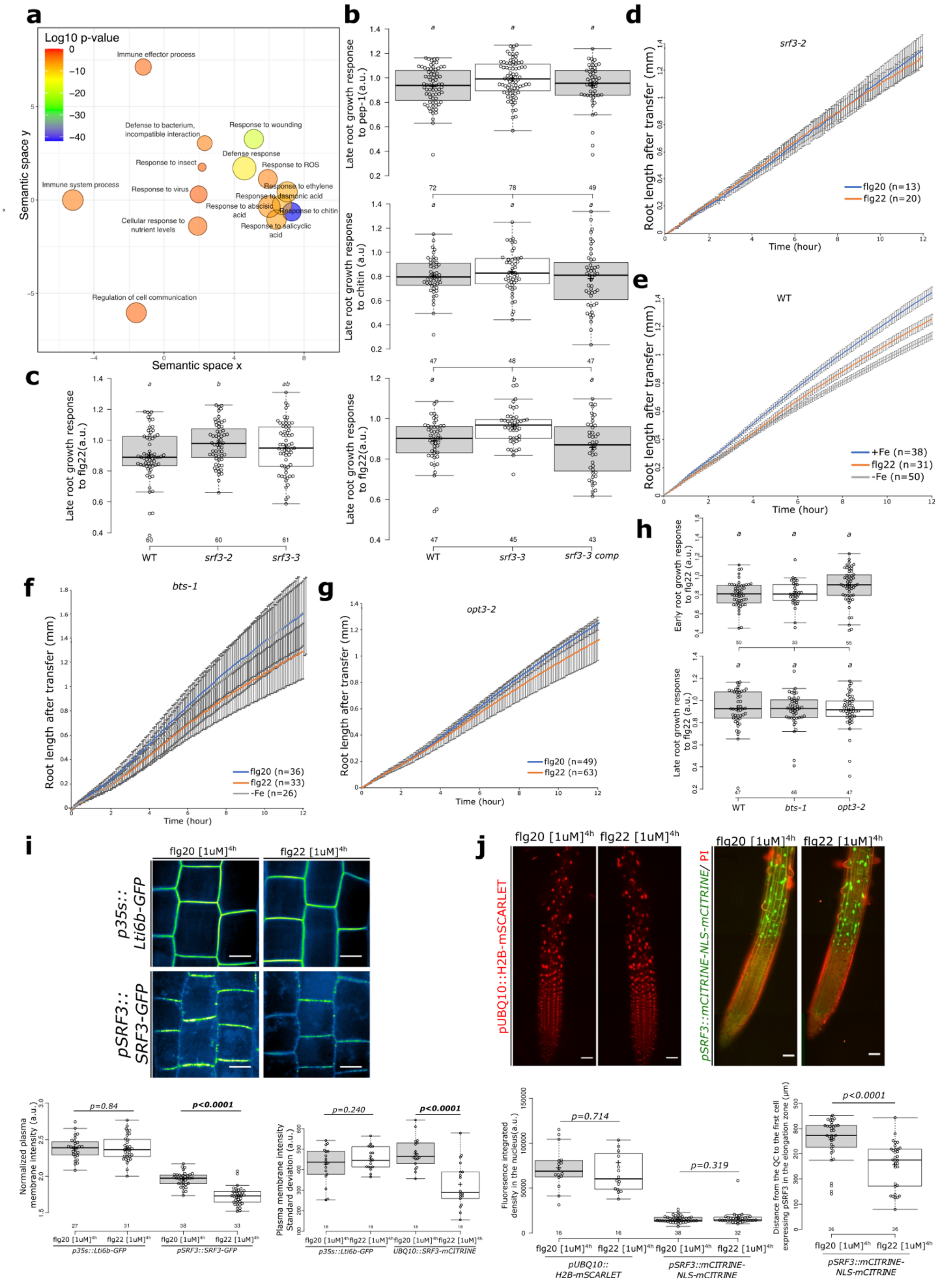

a
Log10 p-value
Immune effector process
Defense to bacterium, incompatible interaction
Response to wounding
Defense response
Response to insect
Response to ROS
Immune system process
Response to virus
Response to ethylene
Response to jasmonic acid
Response to abscisic acid
Response to chitin
Cellular response to nutrient levels
Response to salicyclic acid
Regulation of cell communication
Semantic space x
Semantic space y
b
Late root growth response to pep-1(a.u.)
Late root growth response to chitin (a.u)
Late root growth response to flg22(a.u.)
WT
srf3-3
srf3-3 comp
c
Late root growth response to flg22(a.u.)
WT
srf3-2
srf3-3
d
srf3-2
Root length after transfer (mm)
flg20 (n=13)
flg22 (n=20)
Time (hour)
e
WT
Root length after transfer (mm)
+Fe (n=38)
flg22 (n=31)
-Fe (n=50)
Time (hour)
f
bts-1
Root length after transfer (mm)
flg20 (n=36)
flg22 (n=33)
-Fe (n=26)
Time (hour)
g
opt3-2
Root length after transfer (mm)
flg20 (n=49)
flg22 (n=63)
Time (hour)
h
Early root growth response to flg22 (a.u.)
Late root growth response to flg22 (a.u.)
WT
bts-1
opt3-2
i
flg20 [1uM]4h
flg22 [1uM]4h
p35s::Lti6b-GFP
pSRF3::SRF3-GFP
Normalized plasma membrane intensity (a.u.)
p=0.84
p<0.0001
Plasma membrane intensity Standard deviation (a.u.)
p=0.240
p<0.0001
p35s::Lti6b-GFP
UBQ10::SRF3-mCITRINE
j
flg20 [1uM]4h
flg22 [1uM]4h
pUBQ10::H2B-mSCARLET
pSRF3::mCITRINE-NLS-mCITRINE/ PI
Fluorescence integrated density in the nucleus(a.u.)
p=0.714
p=0.319
pUBQ10:: H2B-mSCARLET
pSRF3::mCITRINE-NLS-mCITRINE
Distance from the QC to the first cell expressing pSRF3 in the elongation zone (µm)
p<0.0001
pSRF3::mCITRINE-NLS-mCITRINE

Supplementary Figure 9. Specific regulation of flg22-induced bacterial root innate immunity responses by SRF3. (a) Visualization of Gene ontology analysis of differential expressed genes in *srf3* mutants compared to the WT under standard growth condition. **(b)** Box plot showing the late root growth responses to pep-1 (top panel), chitin (middle panel) and flg22 (bottom panel) in WT, *srf3-3*, *srf3-3* complementation lines. One-way ANOVA followed by a post-hoc Tukey HSD test, letters indicate statistical differences ($p<0.05$). (**c)** Box plot showing the late root growth response to flg22 in WT, *srf3*-2 and *srf3-3*. One-way ANOVA followed by a post-hoc Tukey HSD test, letters indicate statistical differences ($p<0.05$). (**d-g)** Time lapse analysis for 12 h of root growth in response to flg22 and iron levels. Error bars indicate standard error of the mean (SEM). (**d**) WT and *srf3-2* under 1 µM of flg20 and flg22, **(e)** WT under iron sufficient and low iron media and media supplemented with 1 µM flg22, **(f)** *bts-1* under flg20 and flg22 at 1µM, **(g)** *opt3-2* under iron deficiency, flg20 and flg22 at 1µM. **(h)** Box plot of early (12 hours; top panel) and late (3 days; bottom panel) root growth response to flg22 of WT, *bts-1* and *opt3-2* . One-way ANOVA followed by a post-hoc Tukey HSD test, letters indicate statistical differences ($p<0.05$). **(i)** Top panel: Confocal images of epidermal cells in root tips of 5 days old seedling expressing *p35s::Lti6b-GFP* and *pSRF3::SRF3-GFP* (*Ler* background) under flg20 and flg22 at 1 µM for four hours. Scale bars, 10µm. Bottom left panel: related quantification of the normalized plasma membrane intensity. Bottom right panel: quantification of the standard deviation of the signal intensity at the apical basal side of the PM in *p35s::Lti6b-GFP* and *pUBQ10::SRF3-mCITRINE* under flg20 and flg22 at 1µM for four hours. Independent two-ways student test ($p<0.05$), n.s. non-significant. **(j)** Upper panel: confocal images of max z-projection of root tips of 5 days old seedling expressing *pUBQ10::H2B-mSCARLET* and *pSRF3::mCITRINE-NLS*-mCITRINE and cells were stained by propidium iodide (PI) under flg22 at 1 µM for four hours, scale bars, 50µm, the related quantification of the nuclear fluorescence integrated density (bottom left) and of the distance from the QC to the cell expressing pSRF3 in the elongation zone in µm (bottom right). Independent two-ways student test ($p<0.05$), n.s. non-significant. For boxplots, circles indicate a single biological replicate and the number below each box specifies the number of replicates, horizontal black bars indicate the median, the black cross represents the mean, box represents the interquartile range and the hinges the min and max whiskers.

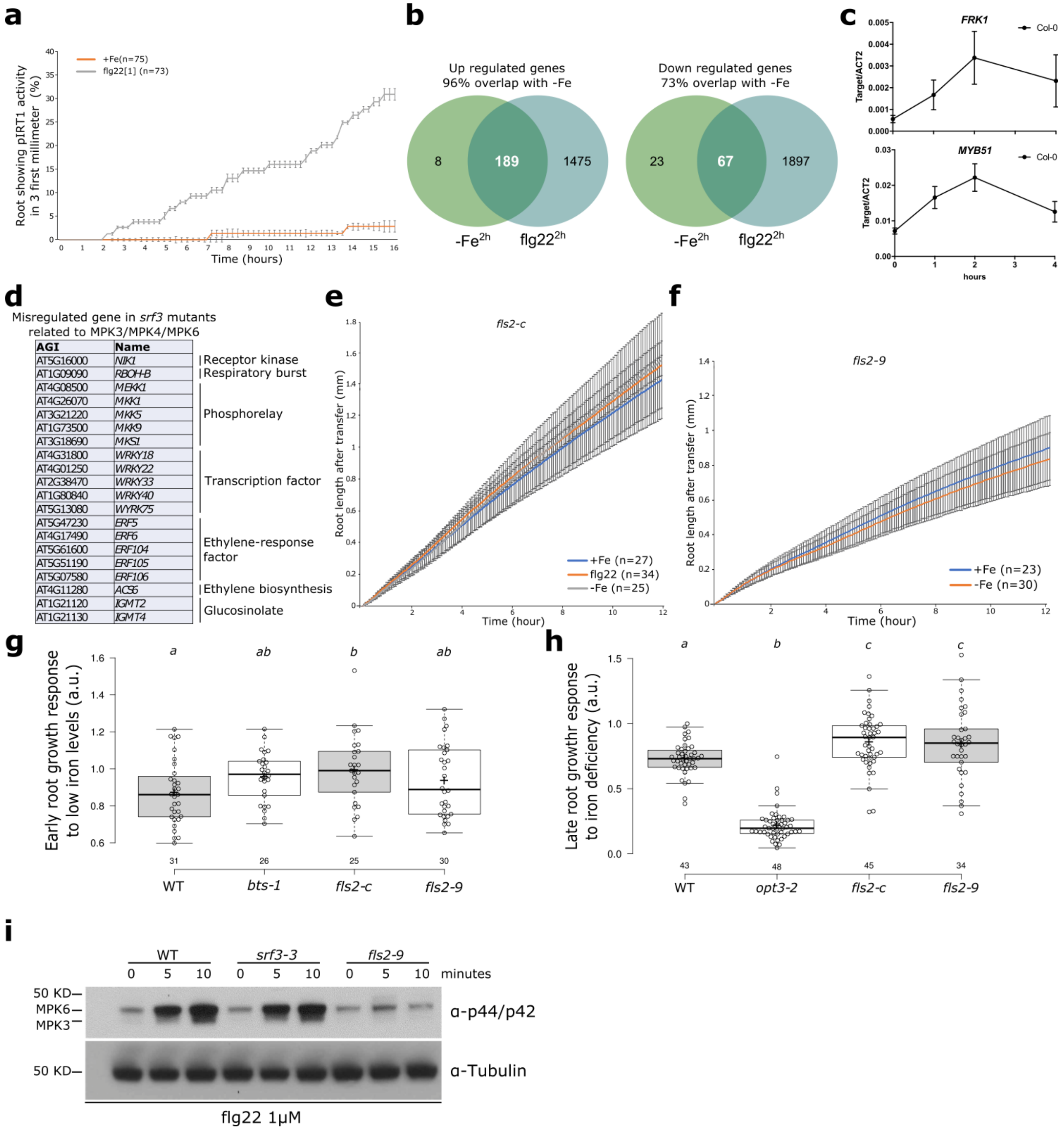
a
+Fe(n=75)
flg22[1] (n=73)
Root showing pIRT1 activity in 3 first millimeter (%)
Time (hours)
b
Up regulated genes
96% overlap with -Fe
8
189
1475
-Fe2h
flg222h
Down regulated genes
73% overlap with -Fe
23
67
1897
c
FRK1
MYB51
Col-0
Target/ACT2
hours
d
Misregulated gene in srf3 mutants related to MPK3/MPK4/MPK6
AGI | Name
AT5G16000 | NIK1 | Receptor kinase
AT1G09090 | RBOH-B | Respiratory burst
AT4G08500 | MEKK1 | Phosphorelay
AT4G26070 | MKK1
AT3G21220 | MKK5
AT1G73500 | MKK9
AT3G18690 | MKS1
AT4G31800 | WRKY18 | Transcription factor
AT4G01250 | WRKY22
AT2G38470 | WRKY33
AT1G80840 | WRKY40
AT5G13080 | WYRK75
AT5G47230 | ERF5 | Ethylene-response factor
AT4G17490 | ERF6
AT5G61600 | ERF104
AT5G51190 | ERF105
AT5G07580 | ERF106
AT4G11280 | ACS6 | Ethylene biosynthesis
AT1G21120 | IGMT2 | Glucosinolate
AT1G21130 | IGMT4
e
fls2-c
+Fe (n=27)
flg22 (n=34)
-Fe (n=25)
Root length after transfer (mm)
Time (hour)
f
fls2-9
+Fe (n=23)
-Fe (n=30)
g
Early root growth response to low iron levels (a.u.)
a ab b ab
31 26 25 30
WT bts-1 fls2-c fls2-9
h
Late root growthr esponse to iron deficiency (a.u.)
a b c c
43 48 45 34
WT opt3-2 fls2-c fls2-9
i
WT srf3-3 fls2-9
0 5 10 0 5 10 0 5 10 minutes
50 KD
MPK6
MPK3
α-p44/p42
α-Tubulin
flg22 1μM

Supplementary Figure 10. SRF3 is involved in the regulation of PTI signaling pathway. (a) Time lapse analysis of pIRT1::NLS-2xYPet under mock and 1 µM flg22 at treatment for 16 hours. Error bars indicate standard error of the mean (SEM). **(b)** Venn diagram of differentially expressed genes, up regulated and down regulated, under iron deficiency and flg22 for 2 hours at 1µM. DEG criteria are as according to the FDR<0.05 **(c)** Expression levels of immune-related genes, *FRK1* (top) and *MYB51* (bottom) under low iron levels over time. Error bars indicate SEM. **(d)** List of known PTI-dependent genes that are mis-regulated in *srf3* mutants which have been found as mis-regulated upon -Fe and flg22 for 2 hours. **(e, f)** Time lapse analysis of root length under sufficient (+Fe) and low (-Fe) iron levels media and flg22 at 1uM for 12 hours in *fls2-c* **(e)** and *fls2-9* mutants **(f).** Error bars indicate SEM. **(g,h)** Related quantification at 12 hours (**g**) and three days (**h**) after transfer to -Fe including the *bts-1* and opt3-2 mutants as positive controls. One-way ANOVA followed by a post-hoc Tukey HSD test, letters indicate statistical differences (p<0.05). **(i)** Western blot using α-p44/p42 which evaluates MAPK3/MPK6 phosphorylation status (upper blot) and α-Tubulin (lower blot) under flg22 treatment at 1 µM in WT, *srf3-3* and *fls2-9* after 0, 5 and 10 minutes of treatment. This has been observed 3 times throughout three independent experiments. For boxplots, circles indicate a single biological replicate and the number below each box specifies the number of replicates, horizontal black bars indicate the median, the black cross represents the mean, box represents the interquartile range and the hinges the min and max whiskers.

Quantification of the late root growth response. Plates containing seedlings were scanned from days 5 to 9 after transfer to different media to acquire images for further quantification of the root growth rate per conditions. Plates were scanned using BRAT software[1] each day and were stacked together using a macro in Fiji (Macro_Match_Align). We then calculated the root length for every day per genotype in each condition to evaluate the root growth rate in Fiji using the segmented line. We first calculated the mean of the root growth rate for each day 5 to 6, 6 to 7, 7 to 8, 8 to 9. These values were used to calculate the mean of root growth rate for 3 days. Then, we divided the mean of root growth rate for 3 days to a given media for each plant by the mean of root growth rate for 3 days after transfer to the control media for the entire related genotype. This ratio was used as the late root growth response to low iron levels. Every experiment was repeated twice.

Quantification of the early root growth response. Root length for each seedling was recorded for 12 hours taking a picture every 5 minutes and quantified using a developed Matlab script (Matlab_RootWalker). From these measurements, we ploted the root length from T0 to T12 after transfer. We obtain a curve representing the root length after transfer from which we calculated the area under the curve using the following formula "(Root length T1+Root length T2)/2*(Time T2-Time T1)". Then, we divided the value of the area under the curve after transfer for each plant in each condition by the area under the curve after transfer to the control media for the entire related genotype. This ratio was used as the early root growth response to low iron levels. Every experiment has been repeated three times.

Quantification of the root meristem and cell size. 5 days old seedlings were transferred to iron sufficient or deficient medium (as described in Gruber et al., 2013)[59] that was contained in small chambers used for the early root growth response (Lab-Tek, Chamberes #1.0 Borosilicate Coverglass System, catalog number: 155361). After 12 hours, the cell wall was stained by placing one drop of propidium iodide 15 μM (10 μg/mL in distilled water) on the root tip for 5 minutes. Images were acquired using the stitching mode on the microscope. The cell size was determined using the Cell-O-Type software in the cortex cells[2]. Alternatively, to calculate the meristem size we have calculated the distance between the quiescent center to the first cell which is longer than it is wide in the cortex cell files. The cell length of the third cell after the first cell that is longer than it is wide in the cortex cell files was measured to determine the root cell elongation. Every experiment has been repeated three times.

Measuring signal intensities at the plasma membrane. Confocal images were first denoised using an auto local threshold applying the Otsu method with a radius of 25 and a median filter with a radius of 2 in Fiji[3]. To remove every single bright pixel on the generated-binary image the despeckle function was applied. To obtain plasma membrane skeleton, we detected and removed every intracellular dot using the "Analyze Particles" plugin with the following parameter, size between 0.0001 and 35 000 μm^2 and a circularity between 0.18 and 1. Then, we selected and cropped a zone which only showed a proper plasma membrane skeleton. We created a selection from the generated-plasma membrane skeleton and transposed it to the original image to calculate the plasma membrane intensity. This process has been automated in a Macro (Macro_PM_Intensity). The plasma membrane intensity value was then divided by the total intensity of the image to normalize the plasma membrane intensity. An average of 45 cells were used for quantification per root. Every experiment was repeated three times.

Calculating standard deviation measures of the intensities at the plasma membrane. The standard deviation of the apical-basal plasma membrane was calculated using the segmented

line tool in the Fiji toolbox using a width of 3 pixels. 5 plasma membranes were used per root and the mean was calculated per root. Every experiment has been repeated three times.

Quantification of the fluorescence intensity in the root tip during time lapse experiments. To acquire images, z-stacks with a stepsize of 50 µm were performed coupled with the stitching mode using Fiji. To determine the variation of our translational and transcriptional reporters under different condition, we measured the signal intensity in the root tip over time using Fiji. Prior analysis, confocal images were stitched, and we generate the maximum intensity projection. We drew a region of interest of the same size in the x and y dimensions, corresponding to the width of the root and in length corresponding to the basal meristem, transition and elongation zones. In this region, for each time point we determined the mean grey value. Note that this value is normalized since for each root the same area has been kept between conditions and genotypes. Every experiment was repeated three times.

Quantification of the polarity Index. 5 days old seedlings of transgenic lines were analyzed to determine the "Polarity index" in root tip epidermis. "Polarity index" is the ratio between the fluorescence intensity (Mean Grey Value function of Fiji software) measured at the PM apical/basal side and PM lateral sides (Line width=3). We selected only cells for which the PM at each pole (apical, basal and laterals) were easily viewable and we selected cells that were entering elongation (at least as long as wide, but no more than twice as long as wide). Quantification was conducted in 100 cells over more than 15 independent plants. This Polarity index reveals the degree of polarity of the fluorescent reporters between the apical/basal side and lateral sides of the PM. Every experiment was repeated three times.

Quantification of the integrated Nuclear and fluorescence signal density of transcriptional reporter lines. To acquire images, z-stacks with a stepsize of 50 µm were performed coupled with the stitching mode. Then, we generated the maximum intensity projection for the z-dimension and then binarized the images using the auto local threshold Bernsen with a radius of 15. The despeckle and erode functions were subsequently used to remove background artefacts. The nucleus in this region were selected using the analyze particles function with the settings for size of 15 and 700 μm^2 and for circularity 0.25 to 1.00. The regions selected were used on the original picture for determining the fluorescence intensity in each nucleus. Then the average nuclear integrated density was calculated per root in order to normalize the intensity by the total root area. This process has been automatized in a macro in Fiji (Macro_Nuclear_Signal_Intensity). From the same images, the number of nuclei was calculated and the root area was detected using the plugin "Wavelet a trou" (http://www.ens-lyon.fr/RDP/SiCE/METHODS.html)[4]. The number of nuclei detected was then divided by the area of the respective root to determine the nuclear density per root. This process was automatized using a Fiji macro (Macro_Nuclear_Density). Every experiment was repeated three times.

Quantification of the IRT1 promotor activation in time lapse series. At each time point, roots showing fluorescence signal in the apical 3 mm of the root were counted. Roots that were already showing activation in this zone or showed slight signals at timepoint 0 were removed from the analysis. Then the number of roots that started to show a fluorescent signal after timepoint 0, were divided by the number of total roots observed in this experiment and multiplied by 100 to obtain the percentage of roots showing pIRT1 activation in the apical 3 mm of the root. Every experiment was repeated three times.

Quantification of the distance of signals from the Quiescent Center in the SRF3 transcriptional reporter line. Based on the propidium iodide staining, the Quiescent Center (QC) region was determined by its morphology. Then, using the straight-line option in Fiji, a line was traced from the QC to the first appearance of a clear, bright signal that reported pSRF3 activity. The distance along this line was calculated to determine the distance from the QC to the first cell expressing *pSRF3* in the elongation zone. Every experiment was repeated three times.

Quantification of DRONPA-s diffusion. After activation of the DRONPA-s reporter, the signal intensity using the integrated density of the signal was calculated in the activated cells as well as the adjacent upper and lower cells using Fiji. The signal intensity once again was calculated in the same regions 6 minutes after the activation. To account for photo bleaching, these values were normalized by dividing the DRONPA-s signal intensity after and post bleach in a zone where DRONPA-s was visible in both images. The average of the normalized integrated density in the surrounding cells was calculated, averaging the values obtained in upper and lower cells. Finally, the ratio of the normalized integrated density after and post bleach was calculated by dividing both values to obtain the DRONPA-s normalized fluorescence intensity. Every experiment was repeated three times.

Quantification of pSUC2::GFP diffusion. In order to evaluate the diffusion of the GFP protein, a ROI of about 1800 μm^2 was drawn at the root tip of each root using Fiji. The mean gray value was calculated and divided by the corresponding area to normalize the value. Every experiment was repeated three times.

Quantification of RhoNOx-1 signal intensity. The root area was detected using the plugin "Wavelet a trou" (http://www.ens-lyon.fr/RDP/SiCE/METHODS.html)[4] and then in this area the mean gray value was calculated and divided by the area to obtain the normalized signal intensity. This process has been automatized on a Fiji Macro, Macro_RhoNox-1

Quantification of aniline blue fluorescence intensity. Using Fiji, the mean gray value of 10 plasma membranes in the apical-basal side of the epidermis in the elongation zone was calculated with the segmented line option with 3 pixels wide. The mean of these values was then divided by the mean gray value in the total area where the plasma membrane signal has been calculated in order to normalize the value due to differential strength of the staining. This final value was used for further analysis. Every experiment was repeated three times.

Quantification of the anti-callose antibody fluorescence intensity. Callose deposition was quantified using the Fiji software. Callose fluorescence intensity was measured at the apico-basal cell walls of root epidermal and cortex cells using the segmented line with a width of 3 pixels. A total of 8 to 10 cell walls were measured per roots and used to calculate the average of anti-callose fluorescence intensity per root. Between 5 to 20 roots per transgenic lines and conditions were used in two independent biological replicates were used.

Quantification of Immunogold. The number of gold particles in the EM micrographs were quantified using Fiji software. The number of particles were counted manually in each compartment; e.g. Cytosol, Cell wall, PM and PD; and then reported relative to the surface of cytosol or cell wall (/μm^2), to the length of PM (/μm) and to individual PD. A total of 25 to 50 micrographs were analyzed for each line and conditions. Two biological replicates were used.

Statistics. Each experiment has been repeated independently at least twice, as in every case the same trend has been recorded for independent experiments, the data have been pooled for further statistical analysis. Each sample were subjected to four different normality tests (Jarque-Bera, Lilliefors, Anderson-Darling and Shapiro-Wilk), sample were considered as a Gaussian distribution when at least one test was significant (p<0.05) using Xlstat.

- As a normal distribution was observed a one-way ANOVA coupled with post hoc Tukey test was performed (p<0.05) using Xlstat. Fig.: 1e, 2b, 3a, 5b, 5e, 6b, 6e, and Supplementary Fig. 1d (top), 1e, 1g, 2a (right), 2b, 3d, 4g, 4q, 4r, 5f (left), 6b, 7c, 8a, 8d, 9b, 9c, 9h and 10g,h.

- As a normal distribution was observed at one-way ANOVA coupled with post hoc Fisher test was performed (p<0.05) using Xlstat: Fig. 2e, 4b, 4d, 5f and Supplementary Fig. 1f, 2a (left), 3f, 8c (p<0.1).

- As a normal distribution was observed an independent two-ways student test was performed (p=0.05) using Xlstat: Fig. 1c, 1f, 2c, 4a, 5d, 6c, 6d, Supplementary Fig. 1d (bottom) 1h, 2c, 3c, 5a, 7a,b, 8b and 9i, j.

- As a normal distribution was not observed at two-ways Kruskal-Wallis coupled with post hoc Steel-Dwass-Critchlow-Fligner procedure was performed (p<0.05) using Xlstat. Supplementary Fig. 5f (right) and 6c.

- As a normal distribution was not observed a two-ways Mann-Whitney test was performed (p<0.05) using Xlstat. Fig. 3c coupled with Montecarlo correction and Supplementary Fig. 5b (right panel).

- As a normal distribution was not observed a one-way Wilcoxon signed-rank test / Lower-tailed was performed (p<0.05) using Xlstat. 4c

For time lapse analysis SAS software was used based on a mixed effect model ($p<0.05$) to test the statistical significance. Fig.: 2d and 6b.

For FDR calculation refer to RNAseq section: Fig. 1d and Supplementary Data 2 and 4 (FDR=0.05).

pIRT1 transcriptional reporter line: To generate the transcriptional pIRT1::NLS-2xYPet reporter line, the *IRT1* promoter (2.6 kb) was cloned at *Sal*I and *Bam*HI restriction sites using the primers pIRT1 Sal_F and pIRT1 Bam_R in the pBJ36 vector carrying two in frame copies of the YPet yellow fluorescent protein fused to SV40 nuclear localization signal (kind gift of Dr. Jeff D.B. Long, UCLA). The *pIRT1::2xYPet-NLS* cassette was digested with *Not*I and cloned in the pART27 binary vector[5]. Note that About 20 independent T1 lines were isolated and between three to six representative mono-insertion lines showing strong activation of *IRT1* promoter in the root epidermis upon low iron, as previously described[6], were selected in T2. The entry and destination vectors used are listed in Supplementary Table 7.

SRF3 constructs in *Ler-0*. To generate pSRF3::SRF3g-GFP from *Ler* background, the SRF3 gene and its native promoter (-1492 nt from the transcription start) was amplified by PCR using genomic DNA as template and the primers attB1-SRF3Ler_F and attB2-SRF3Ler_R. The resulting amplicon was purified, sequenced and subcloned into pDONR221 by Gateway BP recombination, following manufacturer's instructions. To generate the C-terminus GFP fusion, the pSRF3::SRF3g fragment was cloned into the binary vector pGWB450 by Gateway LR recombination[7]. The entry and destination vectors used are listed in Supplementary Table 7.

SRF3 constructs (entry vectors). The full-length coding sequence of SRF3 (At4g03390) was amplified by RT-PCR using 7-day old Arabidopsis seedlings cDNA as template and the SRF3_CDS_p221_F and PSRF3_CDS_p221_noSTOP_R primers. The corresponding PCR product was recombined into pDONR221 vector by BP reaction to give SRF3cds-noSTOP/pDONR221. To remove the SRF3 extracellular domain the primers SRF3_kinase_p221_F and SRF3_kinase_p221_R were used to amplify the SRF3 kinase domain. The corresponding PCR product was recombined into pDONR221 vector by BP reaction to give SRF3cdsΔextraC_p221. To remove the SRF3 kinase domain 5' phosphorylated primers were used SRF3_ΔKinase2-5'_F and SRF3_ΔKinase-5'_R followed by a ligation to give SRF3cdsΔKinase_pDONR221. SRF3 mutant impaired in the kinase activity was obtained by site directed mutagenesis using SRF3-cds_mutKD_p221_F and SRF3-cds_mutKD_p221_R to give SRF3cds KDmut_pDONR221. The entry and destination vectors used are listed in Supplementary Table 7.

Promoters and fluorescent proteins (entry vectors). The *SRF3* promoter (5078bp upstream of 5'UTR until the 3'UTR of the previous gene) was cloned using the GIBSON cloning method (https://www.neb.com/applications/cloning-and-synthetic-biology/dna-assembly-and-cloning/gibson-assembly#tabselect3) with the following primers, Insert_pSRF3_F, Insert_pSRF3_R and Backbone_pSRF3_F and Backbone_pSRF3_R introduced into the P4P1R vector (life technologies www.lifetechnologies.com/) to give SRF3prom/pDONR4P1R. The fluorescent mSCARLET protein was synthesized (GeneArt, www.thermofischer.com), amplified with attB2r and attB3 gateway sites using the mSCARLET_F and mSCARLETwSTOP_R primers, and then recombined into pDONRP2R-P3 by BP reaction to yield the mSCARLET/pDONRP2R-P3 entry vector. For allelic complementation the full genomic sequence of *SRF3* extreme accessions have been amplified using the primer gSRF3_F and gSRF3_R and introduce in the destination vector pGreen . The entry and destination vectors used are listed in Supplementary Table 7.

REFERENCES

1. Slovak, R. *et al.* A Scalable Open-Source Pipeline for Large-Scale Root Phenotyping of Arabidopsis. *The Plant Cell* **26**, 2390–2403 (2014).

2. French, A. P. *et al.* Identifying biological landmarks using a novel cell measuring image analysis tool: Cell-o-Tape. *Plant Methods* **8**, 7 (2012).

3. Schindelin, J. *et al.* Fiji: an open-source platform for biological-image analysis. *Nat Methods* **9**, 676–682 (2012).
4. Bayle, V., Platre, M. P. & Jaillais, Y. Automatic Quantification of the Number of Intracellular Compartments in Arabidopsis thaliana Root Cells. *BIO-PROTOCOL* **7**, (2017).
5. Gleave, A. P. A versatile binary vector system with a T-DNA organisational structure conducive to efficient integration of cloned DNA into the plant genome. *Plant Mol Biol* **20**, 1203–1207 (1992).
6. Vert, G. *et al.* IRT1, an Arabidopsis Transporter Essential for Iron Uptake from the Soil and for Plant Growth. *Plant Cell* **14**, 1223–1233 (2002).
7. Nakagawa, T. *et al.* Development of series of gateway binary vectors, pGWBs, for realizing efficient construction of fusion genes for plant transformation. *J Biosci Bioeng* **104**, 34–41 (2007).
8. Hellens, R. P., Edwards, E. A., Leyland, N. R., Bean, S. & Mullineaux, P. M. pGreen: a versatile and flexible binary Ti vector for Agrobacterium-mediated plant transformation. *Plant Mol Biol* **42**, 819–832 (2000).
