# Supplementary material for: The receptor kinase SRF3 coordinates iron-level and flagellin dependent defense and growth responses in plants
Source: Nat Commun. 2022 Aug 1;13:4445. doi: 10.1038/s41467-022-32167-6 (PMC9343624; doi:10.1038/s41467-022-32167-6)
Supplement: Supplementary file 3 — Description of Additional Supplementary Files [file 41467_2022_32167_MOESM3_ESM.pdf]

Description of Additional Supplementary Files

File Name: Supplementary Data 1
Description: Accessions used in this study and their root growth rate (RGR) values (in mm) and GWAS peaks.

File Name: Supplementary Data 2
Description: Mis-regulated genes in srf3-3 mutants compare to WT in +Fe conditions.

File Name: Supplementary Data 3
Description: Identification of mis-regulated iron deficiency-related genes upon early defense response.

File Name: Supplementary Data 4
Description: DEGs under iron deficiency and flg22 for 2 hours and their overlap.

File Name: Supplementary Data 5
Description: SRF3-dependent early Iron/defense core regulatory network.

File Name: Supplementary Data 6
Description: Primers.

File Name: Supplementary Data 7
Description: Entry and destination vectors used in the study.

File Name: Supplementary Movie 1
Description: Time lapse of WT plants under iron sufficient and low iron conditions for 12 hours.

File Name: Supplementary Movie 2
Description: Time lapse of srf3-3 plants under iron sufficient and low iron conditions for 12 hours.

File Name: Supplementary Movie 3
Description: Time lapse of 5 day old plants expressing pUBQ10:H2B-mSCARLET under iron and low iron for 4 hours.

File Name: Supplementary Movie 4
Description: Time lapse of 5 day old plants expressing pSRF3::mCITRINE-NLS-mCITRINE under iron and low iron for 4 hours.

File Name: Supplementary Movie 5
Description: Time lapse of 5-day old plants expressing pUBQ10:SRF3-mCITRINE under iron and low iron for 4 hours.

File Name: Supplementary Movie 6
Description: Time lapse of 5 day old plants expressing pIRT1::NLS-3xYPet under iron sufficient condition, bright field (left), fluorescence (middle) and plants expressing pUBQ10::H2B-mSCARLET, fluorescence only (right).

File Name: Supplementary Movie 7

Description: Time lapse of 5 day old plants expressing pIRT1::NLS-3xYPet under low iron condition, bright field (left), fluorescence (middle) and plants expressing pUBQ10::H2B-mSCARLET, fluorescence only (right).

File Name: Supplementary Movie 8
Description: Time lapse of 5-day old WT plants under flg20 and flg22 conditions for 12 hours.

File Name: Supplementary Movie 9
Description: Time lapse of 5-day old srf3-3 plants under flg20 and flg22 conditions for 12 hours

File Name: Supplementary Movie 10
Description: Time lapse of 5-day old plants expressing pIRT1::NLS-3xYPet under flg22 condition, bright field (left), fluorescence (middle) and plants expressing pUBQ10::H2B-mSCARLET, fluorescence only (right)
