# Supplementary material for: The receptor kinase SRF3 coordinates iron-level and flagellin dependent defense and growth responses in plants
Source: Nat Commun. 2022 Aug 1;13:4445. doi: 10.1038/s41467-022-32167-6 (PMC9343624; doi:10.1038/s41467-022-32167-6)
Supplement: Supplementary file 21 — Reporting Summary [file 41467_2022_32167_MOESM21_ESM.pdf]

nature portfolio

Corresponding author(s): Wolfgang Busch

Last updated by author(s): Mar 14, 2022

Reporting Summary

Nature Portfolio wishes to improve the reproducibility of the work that we publish. This form provides structure for consistency and transparency in reporting. For further information on Nature Portfolio policies, see our Editorial Policies and the Editorial Policy Checklist.

Statistics

For all statistical analyses, confirm that the following items are present in the figure legend, table legend, main text, or Methods section.

n/a	Confirmed	
☐	☒	The exact sample size (*n*) for each experimental group/condition, given as a discrete number and unit of measurement
☐	☒	A statement on whether measurements were taken from distinct samples or whether the same sample was measured repeatedly
☐	☒	The statistical test(s) used AND whether they are one- or two-sided<br>*Only common tests should be described solely by name; describe more complex techniques in the Methods section.*
☒	☐	A description of all covariates tested
☐	☒	A description of any assumptions or corrections, such as tests of normality and adjustment for multiple comparisons
☐	☒	A full description of the statistical parameters including central tendency (e.g. means) or other basic estimates (e.g. regression coefficient) AND variation (e.g. standard deviation) or associated estimates of uncertainty (e.g. confidence intervals)
☒	☐	For null hypothesis testing, the test statistic (e.g. *F*, *t*, *r*) with confidence intervals, effect sizes, degrees of freedom and *P* value noted<br>*Give P values as exact values whenever suitable.*
☒	☐	For Bayesian analysis, information on the choice of priors and Markov chain Monte Carlo settings
☒	☐	For hierarchical and complex designs, identification of the appropriate level for tests and full reporting of outcomes
☒	☐	Estimates of effect sizes (e.g. Cohen's *d*, Pearson's *r*), indicating how they were calculated

Our web collection on statistics for biologists contains articles on many of the points above.

Software and code

Policy information about availability of computer code

Data collection	Microsof Excel Version 16.42; MatLab 6.5 R13; RStudio 1.3 1093; Fiji 2.1.0/1.53c
Data analysis	MatLab 6.5 R13; RStudio 1.3 1093; Fiji 2.1.0/1.53c; Script for normalized PM intensity (available on github)

For manuscripts utilizing custom algorithms or software that are central to the research but not yet described in published literature, software must be made available to editors and reviewers. We strongly encourage code deposition in a community repository (e.g. GitHub). See the Nature Portfolio guidelines for submitting code & software for further information.

Data

Policy information about availability of data

All manuscripts must include a data availability statement. This statement should provide the following information, where applicable:

- Accession codes, unique identifiers, or web links for publicly available datasets
- A description of any restrictions on data availability
- For clinical datasets or third party data, please ensure that the statement adheres to our policy

https://github.com/mplatre/SRF3_Iron_Defense.git

Field-specific reporting

Please select the one below that is the best fit for your research. If you are not sure, read the appropriate sections before making your selection.

☒ Life sciences ☐ Behavioural & social sciences ☐ Ecological, evolutionary & environmental sciences

For a reference copy of the document with all sections, see nature.com/documents/nr-reporting-summary-flat.pdf

Life sciences study design

All studies must disclose on these points even when the disclosure is negative.

Sample size	To determine the sample size, we did not use any statistical test. We collected between 20-50 independent measurements through 2 or 3 independent experiments.
Data exclusions	For the root growth rate, roots that did not grow for one day (long-term) or for 6 hours (short-term), where removed because they were considered as dead due to the plant transfer with tweezers to new condition. For image obtained by confocal, images selected for analysis correspond to those which do not present mosaic expression of the transgene and which have not been damaged during transfer to the slide.
Replication	To verify the reproducibility, every experiment has been done at least 2-3 times. The most critical one repeated by one or two independent investigators for at least twice repeats. We noticed that during several weeks in summer, the early root growth growth decrease under low iron as measured by microscopy was less pronounced or not measurable. It might therefore be possible that that a season related variable like higher ambient temperature or humidity was responsible for these observations. However, we replicated the root growth experiments in spring, fall and winter and did not observe notable variability between these repeats.
Randomization	N.A.
Blinding	The critical experiments such as, long and short term root growth response of srf3 mutants and genetic analysis Kinase, ExtraC and CALS3 studies, SRF3 protein decrease under low iron and flg22, were done in double blind. (Santosh Satbhai, Julie Neveu, Matias Gleason, Lukas Brent).

Reporting for specific materials, systems and methods

We require information from authors about some types of materials, experimental systems and methods used in many studies. Here, indicate whether each material, system or method listed is relevant to your study. If you are not sure if a list item applies to your research, read the appropriate section before selecting a response.

Materials & experimental systems

n/a	Involved in the study	
☐	☒	Antibodies
☒	☐	Eukaryotic cell lines
☒	☐	Palaeontology and archaeology
☒	☐	Animals and other organisms
☒	☐	Human research participants
☒	☐	Clinical data
☒	☐	Dual use research of concern

Methods

n/a	Involved in the study	
☒	☐	ChIP-seq
☒	☐	Flow cytometry
☒	☐	MRI-based neuroimaging

Antibodies

Antibodies used	anti-GFP rabbit polyclonal antibody (A11122, Thermo Fisher Scientific); colloidal gold-conjugated goat anti-rabbit IgG (Tebu-Bio); callose antibody (400-2 Australia Biosupplies); anti-GFP horseradish peroxidase-coupled antibodies (Miltenyi Biotech 130-091-833); phospho-P44/42 MAPK antibodies (Cell Signaling, Cat. No. #4370); Goat Anti-Rabbit IgG (H + L)-HRP Conjugate (Bio-Rad, Cat. No. 170-6515)
Validation	All the antibody used here have been commonly used for several decades and validates by the supplier as well as through seversl relevant studies.
